# Brain system segregation and pain catastrophizing in chronic pain progression

**DOI:** 10.3389/fnins.2023.1148176

**Published:** 2023-03-16

**Authors:** Selma Delgado-Gallén, MD Soler, María Cabello-Toscano, Kilian Abellaneda-Pérez, Javier Solana-Sánchez, Goretti España-Irla, Alba Roca-Ventura, David Bartrés-Faz, Josep M. Tormos, Alvaro Pascual-Leone, Gabriele Cattaneo

**Affiliations:** ^1^Institut Guttmann, Institut Universitari de Neurorehabilitació adscrit a la UAB, Barcelona, Spain; ^2^Departament de Medicina, Facultat de Medicina, Universitat Autónoma de Barcelona, Bellaterra, Spain; ^3^Fundació Institut d’Investigació en Ciències de la Salut Germans Trias i Pujol, Barcelona, Spain; ^4^Institut d’Investigacions Biomèdiques August Pi i Sunyer (IDIBAPS), Barcelona, Spain; ^5^Departament de Medicina, Facultat de Medicina i Ciéncies de la Salut, Universitat de Barcelona, Barcelona, Spain; ^6^Centro de Investigación Traslacional San Alberto Magno, Facultad de Medicina y Ciencias de la Salud, Universidad Católica de Valencia San Vicente Mártir, Valencia, Spain; ^7^Hinda and Arthur Marcus Institute for Aging Research and Center for Memory Health, Hebrew SeniorLife, Boston, MA, United States; ^8^Department of Neurology, Harvard Medical School, Boston, MA, United States

**Keywords:** chronic pain, functional magnetic resonance imaging, resilience, system segregation, pain catastrophizing, psychological distress

## Abstract

Pain processing involves emotional and cognitive factors that can modify pain perception. Increasing evidence suggests that pain catastrophizing (PC) is implicated, through pain-related self-thoughts, in the maladaptive plastic changes related to the maintenance of chronic pain (CP). Functional magnetic resonance imaging (fMRI) studies have shown an association between CP and two main networks: default mode (DMN) and dorsoattentional (DAN). Brain system segregation degree (SyS), an fMRI framework used to quantify the extent to which functional networks are segregated from each other, is associated with cognitive abilities in both healthy individuals and neurological patients. We hypothesized that individuals suffering from CP would show worst health-related status compared to healthy individuals and that, within CP individuals, longitudinal changes in pain experience (pain intensity and affective interference), could be predicted by SyS and PC subdomains (rumination, magnification, and helplessness). To assess the longitudinal progression of CP, two pain surveys were taken before and after an in-person assessment (physical evaluation and fMRI). We first compared the sociodemographic, health-related, and SyS data in the whole sample (no pain and pain groups). Secondly, we ran linear regression and a moderation model only in the pain group, to see the predictive and moderator values of PC and SyS in pain progression. From our sample of 347 individuals (mean age = 53.84, 55.2% women), 133 responded to having CP, and 214 denied having CP. When comparing groups, results showed significant differences in health-related questionnaires, but no differences in SyS. Within the pain group, helplessness (β = 0.325; *p* = 0.003), higher DMN (β = 0.193; *p* = 0.037), and lower DAN segregation (β = 0.215; *p* = 0.014) were strongly associated with a worsening in pain experience over time. Moreover, helplessness moderated the association between DMN segregation and pain experience progression (*p* = 0.003). Our findings indicate that the efficient functioning of these networks and catastrophizing could be used as predictors of pain progression, bringing new light to the influence of the interplay between psychological aspects and brain networks. Consequently, approaches focusing on these factors could minimize the impact on daily life activities.

## 1. Introduction

Chronic pain (CP) is an unpleasant sensory and emotional experience associated with negative cognitive and emotional aspects like the feeling of unpleasantness, pain catastrophizing (PC), and decreased physical and psychological functioning across the lifespan (Dahlhamer, 2018). This prolonged pain experience encompasses pain intensity (i.e., how much a patient is in pain) and pain affection aspects, the degree of emotional arousal caused by the sensory experience of pain (Haefeli and Elfering, 2006).

These aspects influence pain perception and modulation, and vice-versa (Wiech, 2016, 2018; Opdebeeck et al., 2018; Wiech and Shriver, 2018; Delgado-Gallén et al., 2021), resulting in large inter-individual differences in terms of pain assessment and treatment. Thus, in the past decades, PC and functional brain connectivity (FC) has been shown to play an important role in understanding pain experience, its chronification, and treatment response.

Pain catastrophizing, intended as the tendency to magnify and ruminate about pain, boasts attentional biases to threatening aspects of painful experience (Gilliam et al., 2017) and, consequently, impacts self-reported pain intensity and pain-related psychological aspects (Jensen et al., 2017; Suso-Ribera et al., 2017; Häggman-Henrikson et al., 2021). Therefore, the combination of PC and unpleasantness can lead to suffering, anger, fear, frustration, or anxiety, having an important role in adjustment to CP (Ziadni et al., 2018) and pain management (Gilliam et al., 2017).

In this line, it has been suggested that the relationship between pain and depressed mood is mediated by PC (Dong et al., 2020). In addition, studies exploring the differential relationships among individual components of PC (helplessness, magnification, and rumination) and pain outcomes (Müller, 2011; Craner et al., 2016; Stensland, 2021), indicated that catastrophic thoughts and behavior are as well linked with pain co-morbidities as sleep (Abeler et al., 2020), mental health (Dong et al., 2020) and physical activity. Furthermore, the predictive value of PC in pain evolution has been found in several studies. For instance, high levels of PC predicted a decrease in physical activity due to sedentary behavior (Zhaoyang et al., 2020), or higher levels of acute or persistent pain after surgery for knee osteoarthritis (Burns et al., 2015).

Also, the neural correlates of these phenomena have been explored in multiple studies. Altered brain connectivity in pain disorders in task-oriented and resting-state functional magnetic resonance imaging (rs-fMRI) has been shown altered in the presence of acute and CP (Baliki et al., 2014; Kucyi et al., 2014; Yu et al., 2014; Hemington et al., 2016; Pfannmöller and Lotze, 2019; Spisak et al., 2019; You et al., 2021; De Ridder et al., 2022; Solé-Padullés et al., 2022). Crucially it has been suggested that two specific main large-scale brain networks are related to altered pain processing and pain chronification: the dorsoattentional network (DAN), and the default mode network (DMN) (Spreng et al., 2013; Baliki et al., 2014; Becerra et al., 2014; Kucyi et al., 2014; Kilpatrick et al., 2015; Seidler et al., 2015; Franzmeier et al., 2017; Kim et al., 2019; van Ettinger-Veenstra et al., 2019; Jones et al., 2020; De Ridder et al., 2022).

DAN network is prominently involved in goal-directed attention and top-down selection of stimuli and responses, interacting dynamically with the salience network (SN) and control executive network. Concretely, DAN is responsible for the maintenance of spatial priority maps for covert spatial attention, saccade planning, and visual working memory (Vossel et al., 2014). In CP patients it has been found that the functional connectivity of this network, especially concerning its connectivity with other large-scale networks, is consistently altered (Coppola et al., 2019; Mao et al., 2022), and could normalize after pain therapy (Yoshino et al., 2018).

On the other hand, the DMN, which controls self-representational processing, is normally deactivated during task or stimulus exposure, but not in CP patients (Legrain et al., 2009; Seminowicz et al., 2011; Kucyi et al., 2014; De Ridder et al., 2022), exhibiting abnormal DMN resting-state functional connectivity (rs-FC). Alteration in the connectivity of this network has been coupled to several pain types (e.g., low back pain, complex regional pain syndrome (CRPS), chronic widespread pain, or osteoarthritic pain), potentially explaining why pain becomes an integral part of the self. Finally, DMN has been also consistently associated with pain anticipation, pain intensity, and PC (Napadow et al., 2010; Ter Minassian et al., 2012; Loggia et al., 2013; Kucyi et al., 2014; De Ridder et al., 2022).

In this study we aim to use a novel approach in the study of the relationship between CP, PC, and brain networks functional connectivity, using rs-fMRI combined with graph theory methods (i.e., the topological organization of brain networks) (Wang et al., 2010; Ewers et al., 2021). Concretely we will study networks system segregation (SyS) (Wig, 2017), a paradigm based on the idea that effective network functioning is supported by maintaining subnetworks’ segregation while simultaneously allowing integration between them (Wig, 2017), resulting in a brain more adaptable to task demands. Brain networks’ segregation degree has been previously associated with cognitive abilities in healthy adults and patients affected by neurologic diseases (van den Heuvel and Sporns, 2013; Malagurski et al., 2020; Ewers et al., 2021; Riedel et al., 2021).

From our knowledge, this is the first study that explores how SyS, the ratio of rs-FC within brain networks and their connection with the rest of the cortex, could be related to the presence of pain, and the potential role of SyS and pain-related psychological factors in longitudinal changes in the pain experience.

## 2. Materials and methods

### 2.1. Participants and study design

**FIGURE 1:**
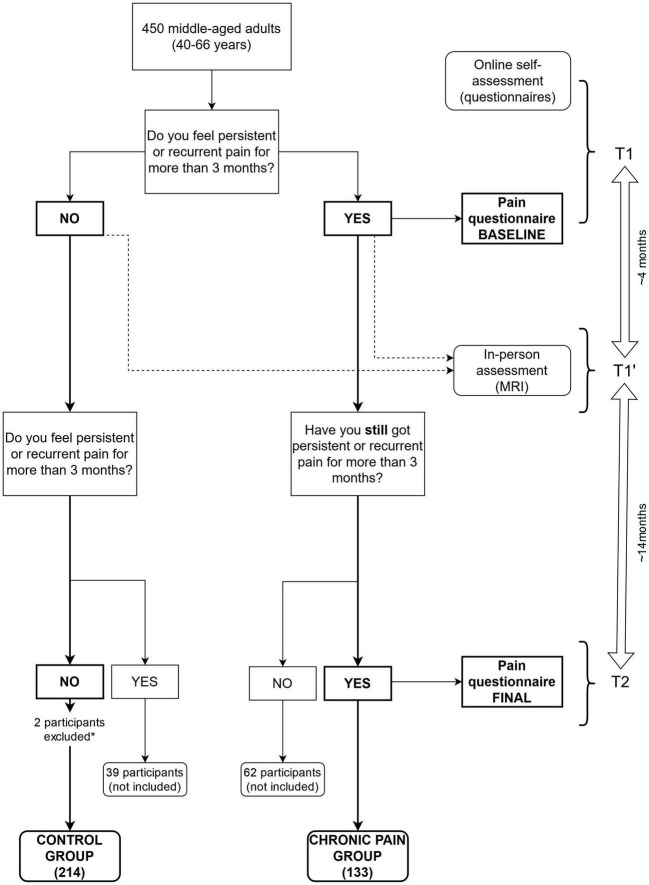
Flowchart of the selection and distribution of patients in this study. Chart illustrating how participants were asked to fill questionnaires (T1 and T2) and undertake an MRI (T1′), and then formed the pain group (YES-YES) and the no pain group (NO-NO). Those participants who did not maintain pain on both questionnaires were not included on this study (YES-NO, NO-YES). Mean time between first questionnaire (T1) and the MRI (T1′) was approximately 4 months, and between the MRI and the second questionnaire (T2), 14 months. *Two participants were excluded from the no pain group for having excessive motion (at least the 50% of the between scans movements were above a 0.5 mm threshold).

This study was performed in the framework of the Barcelona Brain Health Initiative (BBHI) (Cattaneo et al., 2018, 2020), an ongoing prospective longitudinal study that started in 2017 to identify lifestyle factors and biological mechanisms underlying good brain health in middle-aged adults (40–65 years). Between 2018 and 2021 participants underwent an online self-assessment (through the BBHI web-based platform) of sociodemographic characteristics, mental health (MH), quality of life (QoL), and self-perceived cognitive concerns (see Figure 1 for more details). Between 2019 and 2020 they answered the baseline pain questionnaire (T1), and from 2020 to 2021 the second one (T2).

Parallelly, between May 2018 and February 2021 (T1′), participants performed an in-person assessment, including a medical exam and MRI. The same in-person evaluation protocol was applied to all participants.

Online questionnaires and in-person assessments were not paralleled in time and were not equal for all participants. The time gap between T1 and T1′ was approximately 4 months, and between T1′ and T2, 14 months. However, the minimum distance between questionnaires, and between the fMRI scan and T2 was fixed at 3 months to leave room for a reliable pain progression and assure that the criteria for the diagnosis of CP (3 months) were satisfied in each questionnaire independently from the other.

Participants with CP were excluded if (1) pain was due to cancer, fracture, infection, or diagnosis of neurologic or psychiatric disease, (2) made active substance abuse disorder in the past 2 years, (3) uses of prescription opioids exceeding 60 mg morphine equivalents per day, (4) were not suitable for MRI scan and (5) they had excessive head motion during the MRI (at least in the 50% of the scans movements were above a 0.5 mm threshold).

From our sample, 450 participants completed online and in-person assessments (see Figure 1): 133 consistently answered having CP (see below for a CP definition) that persisted for at least 3 months in both questionnaires, 216 without CP in any time-point (where 2 participants were excluded for excessive head motion during MRI), 39 participants that developed CP (that is, they first answered not having CP, and in the second questionnaire they did), and 62 that recovered from a CP (i.e., they started with CP and then they reported not having pain anymore). Considering our principal aim to study pain chronification and its maintenance, the present work included 347 volunteers (133 with CP and 214 without CP).

The protocol was approved by the “Comité d’Ètica i Investigació Clínica de la Unió Catalana d’Hospitals” and was carried out following the Declaration of Helsinki (World Medical Association, 2013). Written informed consent was obtained from all participants before inclusion in the study.

### 2.2. Assessments

#### 2.2.1. Pain: Clinical symptoms

Participants were screened online for CP, which was considered the pain that persists or recurs for > 3 months according to the International Association for the Study of Pain (IASP) criteria (Treede et al., 2015).

Only those participants who answered to have recurring or persisting pain underwent the posterior pain assessment explained below. Thus, the pain group in this study was formed by participants who answered to have pain during at least 3 months in both questionnaires (T1 and T2), while participants who had not CP in either of the questionnaires were included in the no pain group. Non-recurrent or non-persistent pain (i.e., acute pain) was not assessed in this study, neither in healthy nor in CP subjects.

The Brief Pain Inventory-Short Form (BPI-SF) (Keller et al., 2004) was used to assess the intensity and severity of pain and pain onset. Validation studies among CP patients and the published Spanish translation demonstrated good psychometric properties (Badia et al., 2003).

Pain intensity was estimated considering the mean of pain intensity in the last week, considering that asking for a short past period (i.e., 1 week) is more reliable than asking for “current” pain (Haefeli and Elfering, 2006).

Pain interference was estimated through seven domains divided into two subdimensions (with the arithmetic mean): affective (relations with others, enjoyment of life, sleep, and mood) and activity (walking, general activity, and work), according to the BPI user guide (Cleeland and Ryan, 1991). Although sleep is seen as a third domain in some studies, we used it in the affective domain (Miettinen et al., 2019).

Questions about the number of pain sites (one, two, three, or four or more pain sites), and pain medication (non-steroidal anti-inflammatory, anti-migraines…) were added to the questionnaire.

The first and second assessments were conducted during 2019–2020 (T1) and 2020–2021 (T2), respectively, (approximately 10 months between both, see Figure 1).

#### 2.2.2. Pain experience over time: Intensity and affective interference

To calculate the longitudinal progression of the pain experience, we calculated the mean between pain intensity and pain affective interference at each time point:


P⁢a⁢i⁢n⁢e⁢x⁢p⁢e⁢r⁢i⁢e⁢n⁢c⁢e⁢ 1=

$$\frac{P\,a\,i\,n\,i\,n\,t\,e\,n\,t\,i\,s\,t\,y_{T\,1}-P\,a\,i\,n\,a\,f\,f\,e\,c\,t\,i\,v\,e\,i\,n\,t\,e\,r\,f\,e\,r\,e\,n\,c\,e_{T\,1}}{2}$$

P⁢a⁢i⁢n⁢e⁢x⁢p⁢e⁢r⁢i⁢e⁢n⁢c⁢e⁢ 2=

$$\frac{P\,a\,i\,n\,i\,n\,t\,e\,n\,t\,i\,s\,t\,y_{T\,2}-P\,a\,i\,n\,a\,f\,f\,e\,c\,t\,i\,v\,e\,i\,n\,t\,e\,r\,f\,e\,r\,e\,n\,c\,e_{T\,2}}{2}$$

Then we subtracted the numerical value of the first assessment from the second assessment, resulting in a worsening if the final score was positive or an amelioration if it was negative.


P⁢a⁢i⁢n⁢e⁢x⁢p⁢e⁢r⁢i⁢e⁢n⁢c⁢e⁢l⁢o⁢n⁢g⁢i⁢t⁢u⁢d⁢i⁢n⁢a⁢l=

Pain⁢experience⁢ 2-Pain⁢experience⁢ 1

#### 2.2.3. Pain catastrophizing

Pain catastrophizing can be understood as an expansion of maladaptive cognitive response during actual or perceived painful stimuli, comprising negative cognitive and emotional processes. The PC scale is a multidimensional construct that encompasses elements of rumination, magnification, and helplessness. It can be computed by summing responses to all 13 items, and uses a 5-point scale, ranging from 0 (not at all) to 4 (all the time). The total scale score ranges from 0 to 52, with higher scores representing greater catastrophic thinking. Participants are asked to indicate the degree to which they experienced each of the 13 thoughts or feelings when experiencing pain. This scale has been demonstrated to have good psychometric proprieties (Sullivan et al., 1995; García Campayo et al., 2008; Olmedilla Zafra et al., 2013) and it has been validated in the Spanish language.

#### 2.2.4. Health-related questionnaires

We employed the ultra-brief self-reported Patient Health Questionnaire-4 (PHQ-4) to assess MH. The PHQ-4 had four items asking about mood disorder symptoms (two items for depression and the other two items for anxiety) in the past 2 weeks. All items were rated on a four-point scale ranging from 0 (not at all) to 3 (nearly every day). The published Spanish translation also demonstrated good psychometric properties in a validation study (Kocalevent et al., 2014).

To assess the QoL, we used WHO-QoL-AGE, a 13-item questionnaire scored on a 5-point Likert scale. This questionnaire attempts to assess satisfaction with one’s life, living place, general health (i.e., hearing, vision…), daily activities, personal relationships, goal achievements, or economic status (The Whoqol Group, 1998). The published Spanish translation has good psychometric data in a validation study (Lucas-Carrasco, 2012).

We used PROMIS^®^ Cognitive Abilities and Cognitive Concerns scales to assess cognitive troubles. This scale consists of 12-item, extracted from the PROMIS item bank, measuring self-reported cognitive deficits in memory, working memory, attention, processing speed, or cognitive flexibility (Fieo et al., 2016). Each item asked participants to answer “within the last 7 days” using five response options.

### 2.3. MRI acquisition and analysis

#### 2.3.1. MRI acquisition parameters

Magnetic resonance imaging data were acquired in a 3T Siemens scanner (MAGNETOM Prisma) (Siemens Healthcare GmbH, Erlangen, Germany) with a 32-channel head coil, at the Unitat d’Imatge per Ressonància Magnètica IDIBAPS (Institut d’Investigacions Biomèdiques August Pi i Sunyer) at Hospital Clínic de Barcelona, Barcelona. MRI session included accelerated multi-band sequences adapted from the Human Connectome Project and provided by the Center of Magnetic Resonance Research at the University of Minnesota. For all participants, a high-resolution T1-weighted structural image was obtained with a magnetization-prepared rapid acquisition gradient-echo (MPRAGE) three-dimensional protocol, and a total of 208 contiguous axial slices were obtained in ascending fashion [repetition time (TR) = 2,400 ms, echo time (TE) = 2.22 ms, inversion time = 1,000 ms, flip angle = 8°, a field of view (FOV) = 256 mm and 0.8 mm isotropic voxel]. Additionally, a high-resolution 3D SPC T2 weighted acquisition was undertaken (TR = 3,200 ms, TE = 563 ms, flip angle = 120°, 0.8 mm isotropic voxel, FOV = 256 mm). In the same session, they also underwent rs-fMRI multiband (anterior-posterior phase-encoding; acceleration factor = 8) interleaved acquisitions (T2*weighted EPI scans, TR = 800 ms, TE = 37 ms, 750 volumes, 72 slices, slice thickness = 2 mm, FOV = 208 mm). All the MRI images were examined by a senior neuroradiologist (NB) to detect any clinically significant pathology (none found). Then, all the acquisitions were visually inspected before analysis (MC-T and LM-P.) to ensure that they did not contain MRI artifacts or excessive motion.

#### 2.3.2. MRI preprocessing

The rs-fMRI preprocessing pipeline comprised spatial standardization and nuisance correction by making use of functions from FMRIB Software Library (FSL; version 5.0.11)^1^, FreeSurfer (version 6.0)^2^ and Statistical Parametric Mapping (SPM12).^3^ To start with, the first 10 scans were removed to ensure magnetization equilibrium. After that, all images were field inhomogeneity corrected (FSL topup tool), all scans realigned to a reference image (FSL MCFLIRT), and then standardized into native T1-weighted space (SPM Coregister). Finally, normalization (SPM Normalize) of all fMRI images to Montreal Neuroscience Institute (MNI152) standard space was performed to ensure among-subjects comparability. As for nuisance correction, different components were defined and manually removed from the rs-fMRI images by the “fsl_regfilt” tool implemented in FSL. These components correspond to (1) motion regressors of rotation, translation, and their derivatives, as estimated during scans’ realignment, (2) a drift estimated by a discrete cosine transform (DCT) as a low-pass frequency filter (< 0.01), and (3) signals from white matter (WM) and cerebrospinal fluid (CSF). To extract these, CSF and WM masks were obtained from automatic subcortical segmentation of brain volume, based on the existence of an atlas containing probabilistic information on the location of structures (Fischl et al., 2002). This step was part of the FreeSurfer “recon-all” processing stream, which was run with default parameters, except for the addition of the T2 flag for the improvement of pial surfaces reconstruction. Both T1-w and T2-w images were used for processing anatomical information.

As head movement may affect rs-fMRI results (Van Dijk et al., 2012; Power et al., 2013, 2014, 2015), the in-scanner head motion was considered. In this study, the framewise displacement (FWD) mean was calculated for every subject. FWD was computed as in Power et al. (2012), using the vectors of rotation and translation estimated during scans’ realignment as part of the preprocessing pipeline (Power et al., 2012).

#### 2.3.3. Functional magnetic resonance imaging: System segregation

**FIGURE 2:**
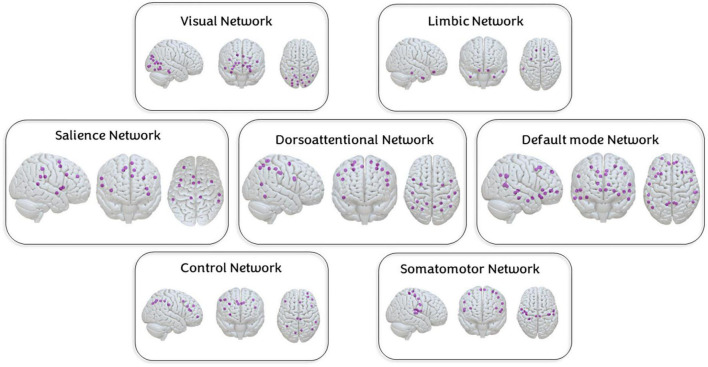
Nodes of the seven networks. Nodes of each of the studied networks based on the Schaffer-Yeo atlas of 100 regions of interest and 7 networks.

A node-based approach was adopted to quantify subject rs-FC and SyS of seven resting-state networks (RSN) as defined in the Yeo atlas (Thomas Yeo et al., 2011). These functional connectivity measures have been previously used to study brain networks implicated in pain progression (Kastrati et al., 2022; Lee et al., 2022). To increase Yeo-atlas spatial resolution and precision at rs-FC computation, the 100 nodes, and 7 networks Schaeffer-Yeo atlas was used (Figure 2; Schaefer et al., 2018).^4^ Per each of the 100 regions of interest (ROIs), a BOLD signal was extracted and averaged across all voxels falling within an ROI. Then, ROI-to-ROI rs-FCs were computed as Pearson-Moment correlations, and subsequently, Fisher-z transformed. Negative values were set to zero and autocorrelations were not considered in the further computation.

**FIGURE 3:**
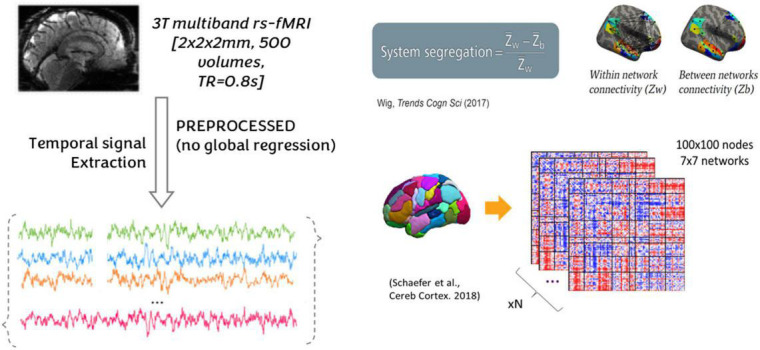
Nodes of the seven networks. A node-based approach was adopted to quantify subject rs-FC and SyS of seven resting-state networks (RSN) as defined in the Yeo atlas (Thomas Yeo et al., 2011). Per each of the 100 regions of interest (ROIs), a BOLD signal was extracted and averaged across all voxels falling within an ROI. Then, ROI-to-ROI rs-FCs were computed as Pearson-moment correlations, and subsequently, Fisher-z transformed. Negative values were set to zero and autocorrelations were not considered in the further computation. SyS captures the balance between within-network (*W*_*net*_) and between-networks (*B*_*net*_) rs-FC. Within-network rs-FC was computed as the average rs-FC connecting all the nodes within the same network. Between-network rs-FC was computed as the average rs-FC connecting nodes of a particular network to nodes from the rest of the cortex.

System segregation (SyS) is a graph theory metric to quantify the extent to which major functional networks are segregated from each other (see Figure 3). As expressed in


$$S\,y\,S_{n\,e\,t}=\frac{W_{n\,e\,t}-B_{n\,e\,t}}{W_{n\,e\,t}},$$

System segregation (SyS) captures the balance between within-network (*W*_*net*_) and between-networks (*B*_*net*_) rs-FC. Within-network rs-FC was computed as the average rs-FC connecting all the nodes within the same network. Between-network rs-FC was computed as the average rs-FC connecting nodes of a particular network to nodes from the rest of the cortex.

### 2.4. Data analysis

#### 2.4.1. Comparisons between no pain and pain groups

We first analyzed group differences (no pain and CP) in sociodemographic data [age, biological sex, and body mass index (BMI)], health-related questionnaire scores (MH, cognitive complaints, and QoL), and head movements during the scan using Welch *t*-tests and chi-square statistics. In addition, we also ran a multivariate analysis between these groups on the segregation of networks, correcting for the delay between the first questionnaire and MRI, head movement during MRI, age, and biological sex.

#### 2.4.2. Correlations between pain variables, psychological aspects, and system segregation

We ran bivariate correlations to explore the association between psychological variables (MH, QoL, cognitive concerns), PC subscales, and pain variables at baseline (intensity, interference, number of pain sites, duration, and medication intake). Besides, we ran partial correlations between SyS and pain factors (intensity, interference, and the number of pain sites), correcting for age, gender, and head motion.

#### 2.4.3. Description of longitudinal pain progression

We explored the differences between pain characteristics (intensity, affective and activity interference, number of pain sites, pain duration, and pain-related medication) in both time points using *t*-tests and chi-square statistics.

#### 2.4.4. Regression models within the chronic pain group

To explore which variables affected pain experience over time, we ran a first multiple regression model with pain experience as the primary outcome, and sociodemographic data (age, biological sex, and BMI), pain baseline ratings (pain-related medication, intensity, total interference, duration, and the number of pain sites), PC scale total score and health-related questionnaires (MH, QoL, and cognitive concerns) as regressors.

Then, we ran different multiple regression models using pain experience progression (Pain experience 2- Pain experience 1) as the dependent variable and sociodemographic (age and biological sex), PC subscales, pain characteristics at baseline (intensity, affective interference, and activity interference), and SyS. We also added covariable head motion during the MRI, as it has an important influence on intrinsic functional connectivity and its interpretation (Van Dijk et al., 2012), in the model and the time between the first questionnaire (T1) and MRI (T1′).

Finally, we added a multicollinearity diagnostic to ensure the interdependence of regressors using the variance inflation factor coefficient. Variables in this step were selected from previous analyses depending on if they had achieved significant effects (like pain intensity, interference, and catastrophizing) or if they could alter SyS results (age, biological sex, distance between first questionnaire and fMRI, and head motion during fMRI).

To explore the interaction between catastrophism’s helplessness and brain networks related to pain progression, we run moderation models through SPSS Process^®^, and we look for the Johnson-Neyman interval. Finally, to explore the possible effect of pain experience in interaction with catastrophism’s helplessness on brain connectivity we repeated the models using brain SyS as the dependent variable and pain experience as the independent variable.

#### 2.4.5. Statistical analyses

Statistical analyses were performed using SPSS version 20.0 (Statistical Package for Social Sciences, Chicago, IL, USA). Regression models’ graphics were created through R v.3.6.3 (R Foundation for Statistical Computing, Vienna, Austria). Brain nodes and segregation graphic representation were created with the Surf Ice tool (version 6-October-2021; v1.0.20211006), which is an OpenGL Shading Language surface rendering source code.

## 3. Results

### 3.1. Differences between no pain and chronic pain group

When we compared the no pain and CP group for sociodemographic data and health-related questionnaires, we found significant differences in age (*F* = 6,271; *p* = 0.013), gender (x^2^ = 8.396; *p* = 0.003), BMI (*F* = 9,352; *p* = 0.002), MH (*F* = 24,772; *p* < 0.001), cognitive complaints (*F* = 31,945; *p* < 0.001), QoL (*F* = 40,392; *p* < 0.001) and head movement during the scan (*F* = 4,652; *p* = 0.032). CP group was composed predominantly of women (63.2%) and older people when compared with the no pain group (mean = 54.81; SD = 7.04) (detailed information is in Table 1).

**TABLE 1:** Sociodemographic data and system network segregation differences between no pain and pain group.

	No pain (214)	Chronic pain (133)	*F*	*p*-value
Sociodemographic	Mean (SD)	Mean (SD)		
Age	52.87 (7.02)	54.81 (7.04)	6,271	0.013*
Biological sex (% women)	47.2%	63.2 %	x^2^ = 8.396	0.003*
BMI	25.36 (3.72)	26.86 (5.40)	9,362	0.002*
**Health-related questionnaires**				
MH	1.21 (1.59)	2.18 (2.02)	24,772	< 0.001**
Cognitive complaints	53.26 (6.54)	48.41 (9.28)	31,945	< 0.001**
QoL	39.90 (7.93)	34.32	40,392	< 0.001**
**Brain networks’ system segregation**				
Default mode	0.268 (0.098)	2.66 (0.095)		0.831
Somatomotor	0.415 (0.082)	0.410 (0.076)		0.871
Control	0.247 (0.084)	0.253 (0.087)		0.380
Dorsoattentional	0.305 (0.067)	0.305 (0.066)		0.533
Salience ventral attentional	0.293 (0.077)	0.304 (0.077)		0.117
Limbic	0.275 (0.104)	0.279 (0.096)		0.822
Visual	0.396 (0.093)	0.393 (0.096)		0.999
Head movement	0.163 (−0.035)	0.179 (0.003)	4,652	0.032*

*< 0.05; **< 0.001. BMI, body mass index; MH, mental health; QoL, quality of life.

Regarding SyS, the multivariate analysis (corrected by head movement, the time between the first questionnaire, biological sex, and age; see Table 1) showed no differences between groups in DMN (*F* = 0.046; *p* = 0.831), somatomotor network (*F* = 0.026; *p* = 0.871), control network (*F* = 0.772; *p* = 0.380), DAN (*F* = 0.390; *p* = 0.533), SN (*F* = 2.466; *p* = 0.117), limbic network (*F* = 0.050; *p* = 0.822) and visual network (*F* = 0.000; *p* = 0.999) (Table 1).

### 3.2. Correlations in psychological aspects and pain variables

Quality of life significantly correlated with multiple pain sites (*r* = −0.243 *p* = 0.005), pain intensity (*r* = −0.282, *p* = 0.001) and interference (*r* = −0.521, *p* < 0.001), cognitive complaints (*r* = 0.398, *p* < .001), MH (*r* = −0.533, *p* < 0.001), rumination (*r* = −0.250, *p* = 0.004), magnification (*r* = −0.442, *p* < 0.001) and helplessness (*r* = −0.478, *p* < 0.001). No correlation was present between QoL and pain duration (*r* = −0.011, *p* = 0.900) or pain medication (*r* = −0.087, *p* = 0.321).

For MH, we found significant correlations with multiple pain sites (*r* = 0.184, *p* = 0.034), pain medication (*r* = 0.187, *p* = 0.031), pain intensity (*r* = 0.213, *p* = 0.014), pain interference (*r* = 0.371, *p* < 0.001), QoL, cognitive concerns (*r* = −0.423, *p* < 0.001), rumination (*r* = 0.288, *p* = 0.001), magnification (*r* = 0.471, *p* < 0.001) and helplessness (*r* = 0.422, *p* < 0.001). No correlation was found with pain duration.

Finally, cognitive complaints were positively correlated with multiple pain sites (*r* = −0.182, *p* = 0.038), MH, QoL, magnification (*r* = −0.222, *p* = 0.011), and helplessness (*r* = −0.191, *p* = 0.029).

Concerning SyS and pain factors (intensity, interference, and the number of pain sites) associations, we only found pain interference and SyS of DMN (*r* = 0.186, *p* = 0.034) and SN (*r* = 0.215, *p* = 0.014), and a tendency of an association with DAN (*r* = 0.161, *p* = 0.068).

### 3.3. Description of longitudinal pain progression

**FIGURE 4:**
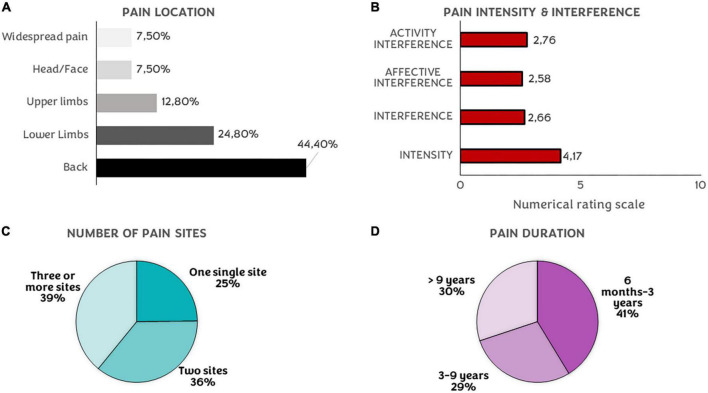
Pain characteristics. **(A)** Percentages of participants’ pain location. **(B)** Mean of interference (sum, activity, and affective) and intensity ratings on a numerical rating scale (0–10). **(C)** Percentages of the number of pain sites. **(D)** Pain duration of the CP in our sample.

Pain characteristics at baseline in our sample are drawn in Figure 4. The most common location was back pain (44.4%), followed by lower (24.8%) and upper limbs (12.8%). During the in-person assessment, participants with CP were asked if they had a diagnosis: 29 suffered from migraine, 15 from cervicalgia, 8 from fibromyalgia, 1 from polymyositis traumatic, 16 from knee pathologies, 54 from low back pain, 15 from other diagnoses that were cursing with CP and 36 had no specific diagnosis. Several participants had more than one diagnosis.

Regarding the number of pain sites, in the first assessment, 75.2% of participants with CP had more than one single painful site and more than half of the sample had their pain for more than 3 years (58.7%). The total interference summed mean was 2.66 (SD = 2.21), 2.76 (SD = 2.36) in the activity subdomain, and 2.58 (SD = 2.39) in the affective subdomain. Finally, the mean intensity for the last week was 4.17 (SD = 1.47).

The pain-relief drugs were the most used treatment at baseline (31.6%), although 31.6% of participants were not enrolled in any kind of treatment at that moment. Finally, 32.3% of our sample treated their pain with other techniques, like physical or psychological therapies. The mean catastrophizing total score was 16.44 (SD = 8.65), helplessness 6.41 (SD = 4.19), rumination 6.44 (SD = 3.37), and magnification 3.60 (SD = 2.26).

When comparing two-time points (Table 2), we found significant differences in intensity (*p* = 0.004), interference (total, affective, and activity) (*p* = 0.021; *p* = 0.033; *p* = 0.047), and medication intake (*p* = 0.024). The number of pain sites and pain duration instead showed no differences (*p* = 0.800). Regarding the worsening or amelioration of pain intensity and affective interference, that is, what we considered as pain experience, 53.3% showed improvements during this period, and 47.7 % exhibited worsening symptoms.

**TABLE 2:** Comparisons between pain variables in first and second questionnaires.

	Pain T1 (mean)	Pain T2 (mean)	*t*	*p*-value
Intensity (last week)	4.17 (1.89)	3.70 (1.95)	2.923	0.004**
Interference total	2.66 (2.21)	2.32 (2.16)	2.332	0.021*
Interference affective	2.59 (2.37)	2.23 (2.22)	2.156	0.033*
Interference activity	2.76 (2.36)	2.44 (2.36)	2.004	0.047*
Number of pain sites	2.35 (1.07)	2.38 (1.063)	−0.254	0.800
Duration (< 3 years) (%)	58.7%	36.8%	0.590	0.556
Duration (3–9 years) (%)	28.6%	36.8%		
Duration (> 9 years) (%)	12.7%	26.4%		
Medication intake (% of yes)	31.6%	42.9%	X^2^ = 5.115	0.024*
Catastrophizing		6.34 (9.64)		
Helplessness		6.41 (4.19)		
Rumination (maximum scoring 16)		6.44 (3.37)		
Magnification (maximum scoring 12)		3.60 (2.26)		

*< 0.05; **< 0.001.

### 3.4. Chronic pain and the role of brain networks in pain progression

When we adjusted the first regression model, where all sociodemographic (BMI, age, and biological sex), health-related questionnaires (MH, QoL, and cognitive concerns), and pain characteristics (PC, pain medication, intensity, interference, duration, and the number of pain sites) were independent variables, we found that PC (β = 0.481; *p* < 0.001), pain intensity (β = −0.299; *p* = 0.002) and total interference (β = −0.562; *p* < 0.001) were associated with pain experience over time.

The second regression model crucially showed that more segregation of DMN (β = 0.193; *p* = 0.037), and less segregation in the DAN (β = −0.215; *p* = 0.014) were associated with worst pain experience progression. Moreover, activity and affective interference (β = 0.255; *p* = 0.022; β = −0.678; *p* < 0.001, respectively), pain intensity at baseline (β = −0.428; *p* < 0.001), and helplessness (β = 0.325; *p* < 0.003) were also related with worst pain experience progression.

**FIGURE 5:**
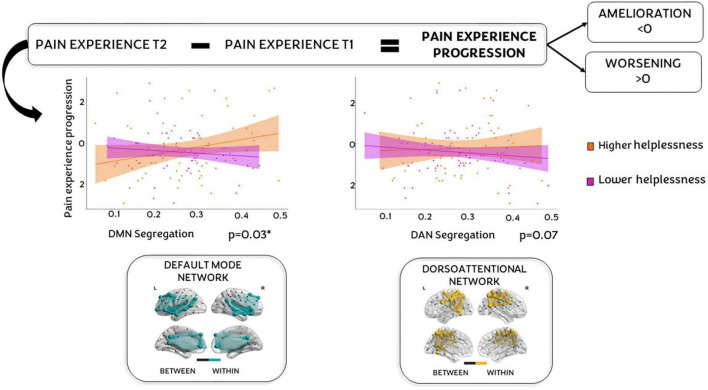
System segregation and helplessness predict chronic pain progression. On top, a chart explaining how pain progression has been calculated (subtracting pain experience T1 from pain experience T2). Positive values indicates a worsening while negative results an amelioration. In the middle, two scatter plots reflecting how each network [default mode network (DMN) at left and dorsoattentional network (DAN) at right] was associated with CP progression depending on low/high helplessness (violet, and orange, respectively). At the bottom of the figure, nodes in the graph represent studied regions of interest (ROIs) as defined by the Schaefer-Yeo atlas of 100 nodes and 7 networks. The nodes and edges illustrate ROIs blue for DMN **(left)** and yellow for DAN **(right)**. Within network connectivity is represented in black (only representative) and refers to outside network ROIs and the connectivity between them and the DAN or DMN.

**FIGURE 6:**
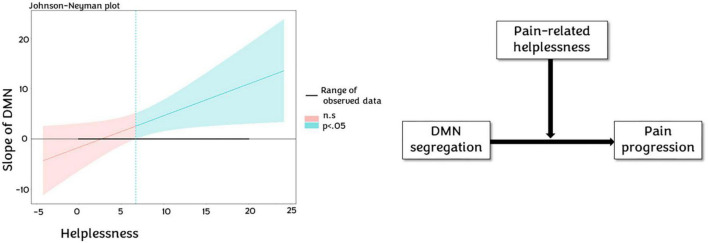
Helplessness moderation. At the **left**, Johnson-Neyman plot show how helplessness moderates the default mode network (DMN) slope since the score 6, where it accentuates the impact of DMN segregation on CP progression. At the **right**, representative chart of moderation of pain helplessness in the relationship between DMN segregation and pain progression.

Finally, when we explored the potential moderator role of helplessness on the relation between SyS and pain experience, we found that helplessness moderates the effect of DMN segregation on pain experience progression (*p* = 0.0313, 5,000 bootstrap samples, 95% CI 0.57–1.20), and a tendency in moderate the relation between DAN SyS (*p* = 0.098, 5,000 bootstrap samples, 95% CI −0.13–1.52) (see Figure 5) and pain experience progression. Johnson-Neyman plots revealed that a score of 7 in the helplessness subscale is the critical value to have a significant association between DMN segregation and pain experience progression (see Figure 6).

When we swap the dependent and independent variables to explore catastrophism’s helplessness, in interaction with pain experience, and modulated brain SyS, we found no significant associations.

## 4. Discussion

In this paper, we explored the role of SyS and psychological aspects in the evolution of pain experience over time. First, significant differences in self-perceived health status were found between the pain and no pain groups. Second, PC and brain network segregation of DMN and DAN were the main predictors of pain progression. Finally, moderation analyses revealed that helplessness, a PC’s subdomain, moderated the relationship between DMN and the progression of the pain experience. To our knowledge, this is the first study using the fMRI SyS model to identify biomarkers that can predict the progression of CP.

### 4.1. Differences between groups and pain characteristics

When we compared participants with and without CP, we found a high prevalence of CP (38.3%) in our sample, according to previous reports of a peak prevalence in late middle-aged adults (50–65 years), affecting up to 20–80% of people (Gibson and Lussier, 2012). The pain group was older, formed predominantly by women, and had an average BMI in the overweight range (bordering on mild obesity), also showing the worst health status (MH, cognition, QoL). This is in line with previous studies that found an association between overweight/obesity and low back pain, particularly stronger in women than men (Breivik et al., 2006; Stone and Broderick, 2012; Dahlhamer, 2018). Another difference observed was an increased head motion during the scan for the pain group, possibly related to an increased difficulty to keep still due to their condition.

We did not observe differences in SyS in any network, probably due to the non-clinical characteristics of our pain sample (i.e., low disability or low impact in their daily lives, low to mild fluctuations in pain intensity, and well-controlled pain).

### 4.2. Pain characteristics and their correlations with psychological aspects

We found several correlations between pain factors and psychological variables (QoL, MH, and self-perceived cognitive complaints), as has been widely described in pain literature (Edwards et al., 2016; Kawai et al., 2017). The subjective and prolonged experience of pain in our cohort was associated with significant mood consequences, such as light depressive symptoms and less capacity to enjoy life. Unquestionably, feeling pain may evolve from normal reactive emotional symptoms, related to stress (Hammen, 2005; Abdallah and Geha, 2017), to clinically relevant depression associated with CP (DeVeaugh-Geiss et al., 2010). Increasing evidence suggests that the pain-related plasticity and depression-related neural circuits are responsible for subtle changes in areas involved in the emotional and cognitive aspects of pain over time that contributes to the behavioral manifestation of altered affective processes (Doan et al., 2015). Finally, as will be discussed later, PC is an important risk factor for a wide range of pain-related effects, including increased pain intensity, increased emotional distress, depression, decreased physical function, and prolonged disability (Schütze et al., 2018).

### 4.3. Catastrophizing is associated with pain progression

Our results indicated that changes in pain experience over time were associated with higher levels of PC and, helplessness was the only domain that vaticinated greater pain impact.

Pain catastrophizing is defined as “an exaggerated negative mental set brought to bear during actual or anticipated painful experience” (Sullivan et al., 2001), and can be divided into three main dimensions: magnification (an exaggerated threat value of pain), rumination (excessive focus pain-related stimuli) and helplessness.

Sullivan (Sullivan, 2012) described how magnification and rumination could overlap with features from primary appraisals (i.e., evaluating the pain stimulus), while helplessness overlaps with secondary appraisal (i.e., the evaluation of oneself to effectively deal with a stressful situation, like pain stimulus). Consequently, cognitions of helplessness are related to higher perceived pain intensity, possibly due to feelings of loss of control, negative future expectations, or rumination, factors that enhance affective pain experience (Müller, 2011).

Pain catastrophizing is related not only to psychological factors but also to altered brain connectivity in pain-related areas and top-down inhibition mechanisms [e.g., a reduced engagement of the descending pain modulatory system; see Malfliet et al. (2017) for review]. Rumination in PC can be considered as a cognitive style (“mind-wandering like”) (Èeko et al., 2015), and is responsible for the occurrence of thoughts not related to a given task and not tied to the immediate environment (Häggman-Henrikson et al., 2021). In general terms, a rumination is a form of circular thinking that swallows the individual in a path without a way out, and it can be broadly defined as a perseverative self-focused thinking process, whereby an individual goes over and over the same thoughts in his or her mind. This process, which can be activated during or after a painful event, generally interferes with a person’s ability to inhibit thoughts, generate alternative ways of thinking, and switch the focus of attention. PC, which possibly appears as a kind of fear of pain, helps perpetuate the cognitive-behavioral pain cycle, collaborating in the activation of negative cognitive and meta-cognitive processes, that in turn can lead to worse coping behaviors and an exacerbation of pain (Ziadni et al., 2018).

This is consistent with previous studies focused on cognitive-affective processes of pain (Gentili et al., 2019; Gonzalez et al., 2019; You et al., 2021). You et al. (2021) demonstrated the existence of pain-specific resilience, referring to the ability to maintain relatively stable and healthy levels of psychological functioning in face of ongoing and persistent pain (You et al., 2021). Sociodemographic (Tanner et al., 2021), structural and functional MRI (Tanner et al., 2021; You et al., 2021), clinical pain symptoms, negative pain-related emotions, and PC (Sturgeon and Zautra, 2013; Gonzalez et al., 2019) have been related to resilience and better outcomes in the presence of recurrent pain, while to our knowledge, this is the first study to explore the interaction between all these variables.

### 4.4. System segregation and pain experience progression

We found associations between pain interference and DMN, and SN, as well as a significant tendency in DAN. One important point, that distinguishes our results from other studies, is that higher segregation of DMN and lower segregation of DAN were associated with this pain experience evolution over time, which is coherent and match with previous results. Besides, helplessness was found to moderate the relationship between DMN and pain experience progression. When we explore the role of the interaction between helplessness and pain experience on brain SyS we found no significant effects, suggesting that brain SyS in certain networks, in interaction with helplessness, affects pain experience evolution over time, and not the reverse.

In this section, we aim to address all the points by substantially revising the main findings on each network individually and collectively, as well as their relationship with helplessness.

First, concerning DAN, its alteration has already been shown to predict pain intensity progression, possibly due to a cognitive evaluation of pain as a permanent threat to the body (Pfannmöller and Lotze, 2019). In this view, our results could be interpreted as dysregulated DAN internal and external connectivity could reduce the ability to correctly process nociceptive stimuli and, consequently, increase the perception of pain intensity over time. Thus, it could be possible that an imbalance in FC between networks causes a sort of “attentional resources kidnapping,” increasing the aversive response to pain. This constant painful input and its maladaptive brain changes (i.e., malfunction of the descending pain pathways), reinforced by catastrophic thoughts, ultimately provoke an increase in self-perceived pain intensity over time.

Similarly, we found a correlation between pain interference and SN segregation, suggesting that, as DAN, altered response in this network could be a contributing factor to the maintenance and chronicity of pain. SN also exhibits task- and resting-state abnormalities in some CP populations (Otti et al., 2013; Becerra et al., 2014; Cauda et al., 2014; Qiu et al., 2021), and may be dysregulated due to constant pain (Borsook et al., 2013), giving rise to a “salient state,” mostly divided into two processes: bottom-up saliency and top-down control (Melloni et al., 2012). Nonetheless, it is well justified that there is an association between a dysregulated SN and the interplay between pain symptoms and psychological aspects, as several studies have also seen before (Legrain et al., 2011; Coppieters et al., 2016; van Ettinger-Veenstra et al., 2019). Indeed, the function of the SN is essential in the processing of sensory stimuli, as the SN plays a key role in the assessment of the inherent danger of such stimuli (and how one should respond to them), and plays a central role in the memory of painful events (Kim et al., 2019).

In the same line DMN alteration in CP patients (Malfliet et al., 2017) has been associated with the “pain state” (Èeko et al., 2020), which facilitates stimulus-independent thoughts, or internally directed, spontaneous or autobiographical thoughts, also resumed in the term “self-generated thought” (Andrews-Hanna et al., 2014). Repetitive negative thinking is considered a form of avoidant coping strategy (Flink et al., 2013). Occupying one’s thoughts with repetitive negative thinking prevents the confrontation with the threat (e.g., What I could have done to prevent this situation?). Additionally, repetitive negative thinking can be negatively reinforced by abstract cognitive activity (e.g., Why do I suffer from pain?). This form of abstract thinking impedes the activation and processing of emotional and somatic responses. The process of suppression/avoidance could magnify the negative emotions and consequently fuel the catastrophic worry cycle. Usually, some of these thoughts are related to personal significance, temporal or social orientation, or somatosensory awareness. Ergo, DMN seems to play an important active role in the self-generation of cognition, thinking about one-self and future thinking, but also with rumination and PC (Èeko et al., 2020).

The predictive value of DMN rs-FC on pain management was already shown by Baliki et al. (2014), where CP disrupted the dynamics of DMN and frontoparietal network (related to attention and working memory), and other regions related to pain modulation (insula; anterior cingulate cortex, ACC) (Baliki et al., 2014). Specific nodes of DMN, like the hippocampus and medial prefrontal cortex (mPFC), are importantly implicated in pain processing. Similarly, Kucyi et al. (2014) demonstrated an enhanced mPFC connectivity in DMN, suggesting its role in the descending modulatory system underlying the degree to which patients ruminate about their CP (Kucyi et al., 2014). Hashmi et al. (2013) also found that strengthening mPFC-nucleus accumbens FC predicts the extension in which brain activity shifts from pain-related to emotion-related regions in patients with persistent subacute back pain, compared to those that get total recovery (Hashmi et al., 2013). In sum, the mPFC can be seen as a hub for the development of mental comorbidities associated with CP. The impaired cholinergic activity contributes to the deactivation of mPFC, possibly leading to cognitive and emotional deficits in CP patients (Kummer et al., 2020).

Overall, our results are consistent with findings reported by Ter Minassian et al. (2012) in their study about brain activity during pain anticipation and pain perception (Ter Minassian et al., 2012). They found that during pain anticipation, DAN was activated while DMN was deactivated, possibly due to the search for strategies to avoid pain. In contrast, during pain perception, DMN was reactivated, whereas DAN remained activated, supporting the fact that DMN and attentional networks cooperate to integrate pain-related stimuli and thoughts, as DMN is at the top of the networks that perform hierarchical integration. The authors concluded that the ACC could be the structure most involved in the coordination between these two networks, given that it can be anatomically and functionally divided during pain anticipation (cognitive; caudal ACC) and pain perception (emotional; rostral ACC).

### 4.5. Default mode and dorsoattentional networks interactions with helplessness

Our results indicate that brain network functioning and cognitive-emotional strategies can interact in modulating pain experience, and both can be understood as possible protectors/risk factors.

Once CP is established, catastrophizing and segregation are not independent factors acting in the pain progression. Segregation of DMN and DAN, indeed, may represent some aspects of brain resilience that could help to maintain or get better from pain, allowing the brain to effectively process pain, or at least avoid areas not involved in pain processing. On the other hand, modifiable psychologic aspects like catastrophizing, related to cognitive reserve, may have a crucial role in pain evolution and the impact of MH. Both kinds of processes can be treated separately but in a coordinated way, as seen in studies of combined behavioral therapies (e.g., pain neuroscience education) with brain stimulation (Meeker et al., 2020; Alcon and Wang-Price, 2022), and could reduce the negative impact of emotions in the evolution of pain over time, collaborating in the selection of effective coping strategies or avoiding negative thinking. Thereby, descending modulation might be more effective, decreasing levels of pain intensity and affective interference. Finally, resilience-focused cognitive-affective approaches, including this holistic view, are important to be applied not only in highly affected CP patients, but also in individuals with a low rating in pain parameters, as they can already show lower QoL, cognition, and MH, or FC alterations.

### 4.6. Limitations and future directions

Our main limitation was that we investigated a non-homogeneous (e.g., different kinds of pain) and non-clinical sample (low ratings in pain intensity, interference, and PC). Thus, in future work, investigating how higher catastrophizing scores, as well as intensity or interference ratings, interact with networks’ segregation in the progression of pain might shed light on how pain alters different networks and how it’s linked to emotional and cognitive aspects.

Another limitation of our study was the lack of information about pain during MRI or the presence of acute pain in the no-pain sample. Further work is certainly required to disentangle these complexities in acute (non-experimentally provoked) and CP. For example, the possibility of prognostic biomarkers of the transition from acute pain to CP, within the framework of pain-related resilience (SyS and psychological factors) is described in this manuscript.

An additional potential limitation was related to time gaps between questionnaires and MRI. Even though we used this gap as a covariable in our analysis, we cannot exclude that this aspect can introduce some bias in the results.

Although we excluded subjects with excess motion and added the mean head motion of each participant as a covariate in all the analyses related to fMRI, we cannot fully dismiss that subject motion to some degree has influenced the present results.

Finally, due to the exploratory nature of our analyses, we did not correct functional connectivity analysis for multiple comparisons.

Future studies need to confirm these results with better controlling for all these aspects and overcome present limitations.

## 5. Conclusion

In summary, present results indicate that non-clinical CP conditions are associated with the segregation of brain networks during rs-fMRI, more concretely SN, DAN, and DMN. Moreover, the latter effect is moderated by helplessness, a catastrophizing domain, that can be seen as a protective factor against pain impact over time.

Our result casts a new light on the interplay among the influence of the interaction between psychological aspects and brain networks in pain management. The extent of the reorganization of these networks and their functioning is critical for the evolution of pain.

Besides, the reorganization of spatial properties of the DMN, DAN, and SN may reflect different emotional, attentional, and cognitive abnormalities observed in CP conditions. Under certain assumptions, this can be construed as the common reorganization among pain patients is the extent of association of the component of the DAN, and its dissociation from the posterior components of the DMN, which seems to disrupt the competitive inhibition between the DMN and the brain networks underlying attention.

Finally, our results highlight the predictive value of fMRI that can orient researchers and clinicians about what form of treatable pathophysiology an individual patient with CP may have. Given that other fMRI techniques are not suitable to measure certain characteristics, such as the effect of attentional fluctuation or the degree of self-generated thoughts, we conclude that SyS networks are an effective metric to understand brain connectivity and neural correlates of the pain state. SyS could be used as biomarkers indicating a predisposition of pain maintenance and allowing disease progression monitoring, as well as the reversion or compensation of these alterations in different kinds of interventions, that should, in the future, be considered for more deep research. We have demonstrated that these variables should be assessed without considering the etiology of pain, giving more importance to the pain state than the pain condition *per se*.

## Data availability statement

The raw data supporting the conclusions of this article will be made available by the authors, without undue reservation.

## Ethics statement

The protocol was approved by the “Comité d’Ètica i Investigació Clínica de la Unió Catalana d’Hospitals” and was carried out following the Declaration of Helsinki (World Medical Association, 2013). Written informed consent was obtained from all participants before inclusion in the study.

## Author contributions

MDS, GC, GE-I, AR-V, MC-T, KA-P, JS-S, AP-L, DB-F, JMT, and SD-G have made substantial contributions to conception, design, and interpretation of data. GC, MDS, and SD-G have made substantial contributions to analysis and interpretation of data. GE-I, AR-V, MC-T, KA-P, JS-S, and SD-G have made substantial contribution to acquisition of data. MDS, GC, and SD-G participated in drafting the manuscript. AP-L, DB-F, JMT, GE-I, AR-V, MC-T, KA-P, and JS-S contributed to revising it critically for important intellectual content. All authors have given final approval of the version to be submitted.

## Footnotes

1
https://fsl.fmrib.ox.ac.uk/fsl/fslwiki/

2
https://surfer.nmr.mgh.harvard.edu

3
https://www.fil.ion.ucl.ac.uk/spm/

4
https://github.com/ThomasYeoLab/CBIG/tree/master/stable_projects/brain_parcellation/Schaefer2018_LocalGlobal
